# Identification of putative drug targets for human sperm-egg interaction defect using protein network approach

**DOI:** 10.1186/s12918-015-0186-7

**Published:** 2015-07-18

**Authors:** Soudabeh Sabetian, Mohd Shahir Shamsir

**Affiliations:** Department of Biosciences and Health Sciences, Faculty of Bioscience and Medical Engineering, Universiti Teknologi Malaysia, Johor, Malaysia

**Keywords:** Protein-protein interactions, Sperm-egg interaction, Essential proteins, Potential drug targets

## Abstract

**Background:**

Sperm-egg interaction defect is a significant cause of *in-vitro* fertilization failure for infertile cases. Numerous molecular interactions in the form of protein-protein interactions mediate the sperm-egg membrane interaction process. Recent studies have demonstrated that in addition to experimental techniques, computational methods, namely protein interaction network approach, can address protein-protein interactions between human sperm and egg. Up to now, no drugs have been detected to treat sperm-egg interaction disorder, and the initial step in drug discovery research is finding out essential proteins or drug targets for a biological process. The main purpose of this study is to identify putative drug targets for human sperm-egg interaction deficiency and consider if the detected essential proteins are targets for any known drugs using protein-protein interaction network and ingenuity pathway analysis.

**Results:**

We have created human sperm-egg protein interaction networks with high confidence, including 106 nodes and 415 interactions. Through topological analysis of the network with calculation of some metrics, such as connectivity and betweenness centrality, we have identified 13 essential proteins as putative drug targets. The potential drug targets are from integrins, fibronectins, epidermal growth factor receptors, collagens and tetraspanins protein families. We evaluated these targets by ingenuity pathway analysis, and the known drugs for the targets have been detected, and the possible effective role of the drugs on sperm-egg interaction defect has been considered. These results showed that the drugs ocriplasmin (Jetrea©), gefitinib (Iressa©), erlotinib hydrochloride (Tarceva©), clingitide, cetuximab (Erbitux©) and panitumumab (Vectibix©) are possible candidates for efficacy testing for the treatment of sperm-egg interaction deficiency. Further experimental validation can be carried out to confirm these results.

**Conclusion:**

We have identified the first potential list of drug targets for human sperm-egg interaction defect using the protein interaction network approach. The essential proteins or potential drug targets were found using topological analysis of the protein network. These putative targets are promising for further experimental validation. These study results, if validated, may develop drug discovery techniques for sperm-egg interaction defect and also improve assisted reproductive technologies to avoid *in-vitro* fertilization failure.

**Electronic supplementary material:**

The online version of this article (doi:10.1186/s12918-015-0186-7) contains supplementary material, which is available to authorized users.

## Background

Sperm-egg interaction is one of the most significant processes in the fertilization event, and the disorder in this process is the main cause of fertilization failure in common *in-vitro* fertilization (IVF) cases with unexplained male infertility [1, 2]. The molecular interaction process surrounding sperm-oocyte binding and fusion have been studied in various attempts over the past 20 years and is still poorly understood. Due to detection of the molecules that immediate human membrane sperm-oocyte interaction, different experimental methods have been carried out [3]. In our previous research (refer to [4, 5]), we have represented that in addition to experimental techniques, computational methods, protein interaction network approach, can address protein-protein interactions between human sperm and egg; we have shown the links between the sperm and egg proteins in the form of a protein interaction network. Several research questions associated with the function of single or groups of interacting proteins can be answered with the help of protein-protein interaction (PPI) networks [6].

Essential proteins for a biological event play a complex role in the development of the process, and the discovery of their features is an interesting research subject in proteomics. A protein’s essentiality has been used in numerous medical and biological researchers in recent years. Currently essential proteins are recognized based on gene knockout experiments, which can be expensive and time consuming when the biological experiments are carried out on a large-scale basis. Therefore, researchers still lack understanding regarding a large number of proteins’ lethality information; thus, alternative detection approaches are being pursued. Computational methods can supply the knowledge of biological information. The absence or dysfunction of essential proteins would create an adverse disruption to the topological stability of the network as in the case of PIN biological lethality. This laid the foundation in which computation methods based on topological features are developed to better detect essential proteins [7–11]. Recent experimental protein interaction networks of *Saccharomyces cerevissiae* and *Caenorhabditis elegans* [8, 12] have been carried out to confirm the effectiveness of topological metrics in predicting protein essentiality, demonstrating strong correlation with knockout and knockdown data.

Until now, no drugs have been identified to treat infertile cases who have a disorder of the sperm-egg interaction process. The drug discovery process begins with a search for drug targets or essential proteins. Because of the networked nature of protein function, topological analysis of the protein network may help to identify essential proteins that can be potentially drug targets.

In this work, we analyzed the human sperm-egg protein interaction network with topological metric of connectivity, betweenness centrality in order to identify essential proteins as putative drug targets using Cytoscape 2.8. Ingenuity pathway analysis (IPA) was used to identify the drugs for these targets to determine the possible effectiveness of these drugs on sperm-egg interaction disorder. The list of putative protein targets is a starting point for experimental validation by *in vitro* assays and further discovery of new drugs in order to treat the sperm-egg interaction defect.

## Methods

### Construction of sperm-egg protein-protein interaction network

Information found in PPIs databases supports the construction of interaction networks. All retrieved data from the various data sources (refer to [4, 5]) were combined and loaded into Cytoscape 2.8 [13] to construct the protein interaction network (PIN). Two PINs for sperm proteins and oocyte proteins have been created, and then intersection (overlapping) network has been constructed using a network modification plugin (compare two networks) in Cytoscape. Because our primary aim was to identify protein candidates involved in sperm-egg interactions, we extracted only the proteins that contain a signal sequence and/or transmembrane domain [14] by selecting signal peptide and transmembrane features from sequence annotation (features) in an extensively curated UniProt database (www.uniprot.org) (for more details refer to [4, 5]).

### Selecting high confident protein interactions

In this study, only the high confidence protein-protein interactions (STRING scores ≥ 0.700 and MINT-inspired (MI) scores ≥ 0.431) were filtered. STRING (string-db.org) uses a score combiner based on the product of probabilities using the following formula: $S=1-{\displaystyle \prod_i^N}\ \left(1-{S}_i\ \right)$, with *S*_*i*_ the probability score for database *i*, *S* the combined score and *N* the total number of databases to be combined. The combined scores were further rescaled into the confidence range from 0.0 to 0.1 combining all the scores. Those indicate: <0.400 (low confidence), 0.400–0.700 (medium confidence) and >0.700 (high confidence). As a result, the high confidence PPI’s (>0.700) were selected [15]. The MINT-inspired (MI) score was assigned based on an earlier approach [16] and it was adopted their confidence score formula (http://wodaklab.org/iRefWeb/). To create a high-confidence set of interactions, the following procedure was applied; first include the physical interactions; then exclude interactions that are supported by less than three publications or that are not conserved in any species and finally retain pairs with an MI-score of at least 0.431 [17]. To avoid a false positive result of experimental detection for physical interactions and to create a high confidence set of physical interactions, the pairs of interactions with an MI- score of at least 0.431 remain [17].

### Identification of putative drug targets with topological analysis

Topological parameters have been defined to measure network characteristics using Cytoscape Network Analyzer plug-in v. 2.8. This plugin is a tool that helps to study the topology and the parameters of a network by using descriptive statistics and graphs. Power law fit for the protein network was detected by calculating degree exponent distribution and the coefficient of determination. The shortest path (geodesics) was calculated to evaluate the network and parameters, such as degree (connectivity), betweenness centrality (BC) and closeness centrality (CC), were used to detect the essential proteins as putative drug targets.

### Detection of the drugs for the putative drug targets

The name of the essential proteins or the identified putative drug targets were submitted in IPA software (http://www.ingenuity.com/), and using disease view feature of IPA, the known drugs for these targets have been detected. We have also demonstrated the hypothetical effectiveness of the drugs on sperm-egg interaction disorder.

## Result and discussion

### Construction of sperm-egg interaction network

**Fig. 1:**
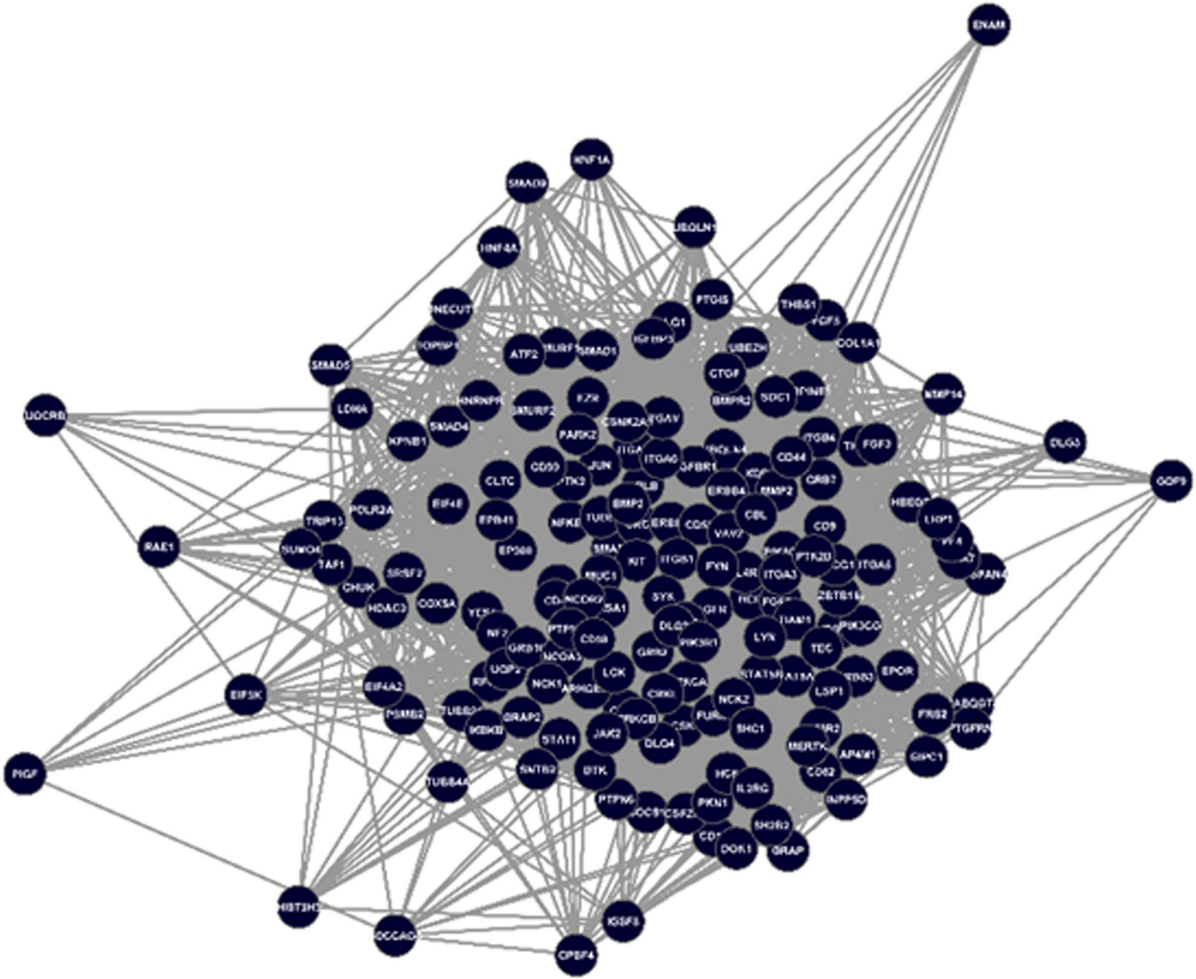
Sperm-Egg Protein Interaction Network. The intersection (overlapping) network between sperm and oocyte PIN was calculated using a network modification plugin (compare two networks) in Cytoscape 2.8. From these proteins, 106 proteins that contain a signal sequence and/or transmembrane domain were extracted. The created protein network consists of 106 nodes and 1484 interactions

In order to construct a sperm-egg interaction network, the sperm protein network including 2076 protein nodes and 8565 interactions and an oocyte protein interaction network including 409 protein nodes and 2746 interactions has been created. Then, the intersection (overlapping) network was calculated using a network modification plugin (compare two networks) in Cytoscape 2.8. The overlapping protein interaction network, namely the sperm-egg interaction network, consists of 167 nodes and 3634 interactions. From these proteins, 106 proteins that contain a signal sequence and/or transmembrane domain were extracted. A variety of databases that are based on the used features and the number of released PPI in sperm-egg interaction from these databases are detailed in the Additional file 1: Table S1 and S2. The extracted protein network as shown in Fig. 1 consists of 106 nodes and 1484 interactions.

### Selecting high confidence predicted protein interactions

In this study, to avoid false positive results of predicted PPIs, the “combined score” between any pair of proteins was calculated using STRING database (string-db.org). As a result, only the interactions in the human sperm-egg interaction network with high confidence (>0.700) PPIs were filtered. To elude false positive results of experimental detection for physical interactions and to create a high confident set of physical interactions, the pairs of interactions that are supported by at least three publications and that have an MI-score of at least 0.431 remain (http://wodaklab.org/iRefWeb/).

**Fig. 2:**
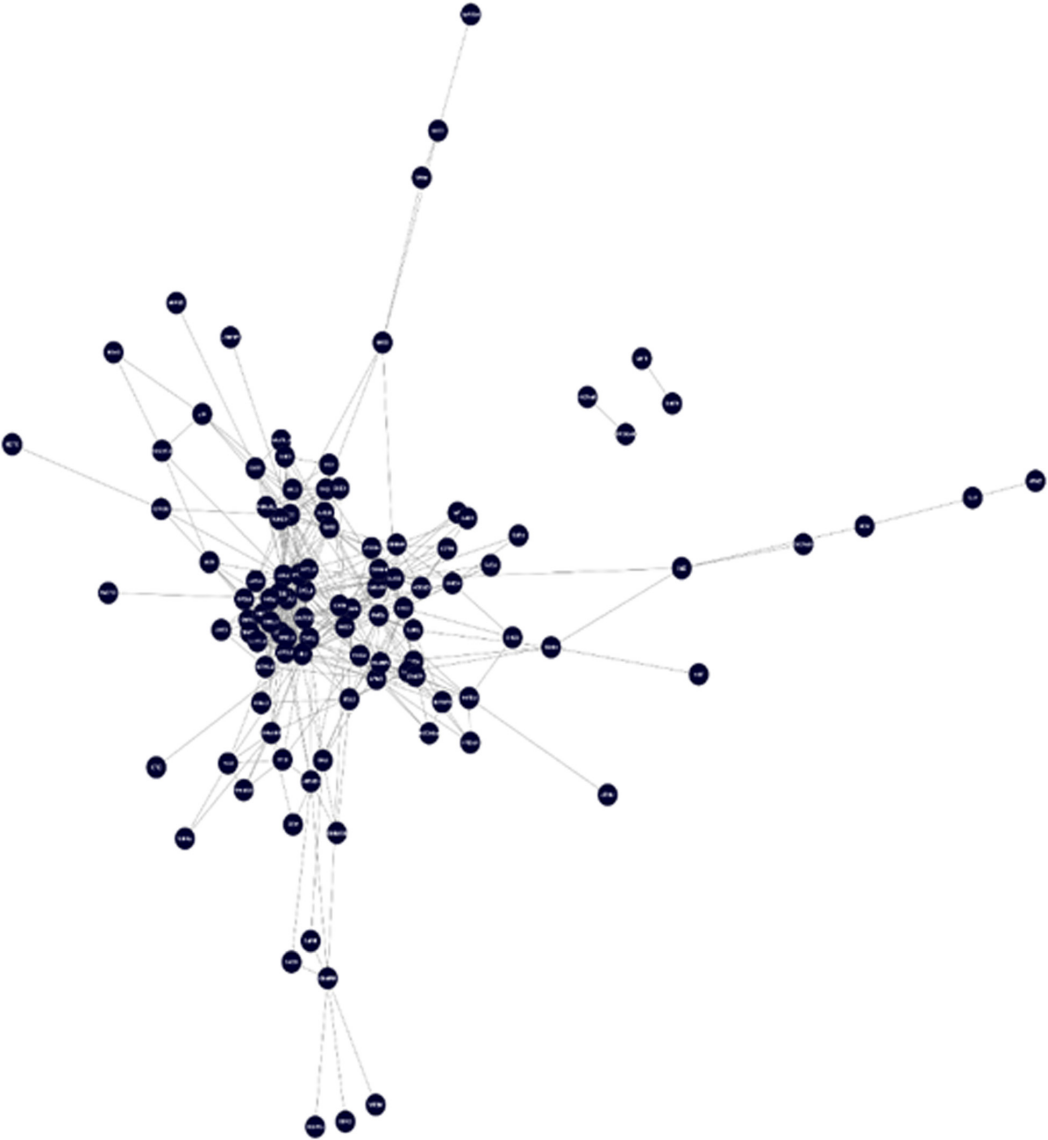
The Sperm-Egg Interaction Network with High Confident PPI Based on STRING Scores and MI Scores. Two created networks with high confident PPIs were merged to achieve a union network. The retrieved network includes 106 nodes and 415 edges

After extracting the high confident PPIs, there were two networks: the network with high confident PPI based on STRING scores that includes 96 proteins and 362 interactions and the physical interaction network with 90 proteins and 186 interactions, as summarized in Table 1. Two created networks were merged to achieve a union network. The retrieved network includes 106 nodes and 415 edges (See Fig. 2).

**Table 1:** The high confident PPI in sperm-egg interaction network

Type of interactions	Proteins	PPI
High confident PPI (>0.700) based on STRING scores	96	362
High confident physical interactions (≥0.431) based on MI scores	90	186

After extracting the high confident PPIs, there were two networks: the network with high confident PPI based on STRING scores that includes 96 proteins and 362 interactions and the physical interaction network with 90 proteins and 186 interactions

### Topological analysis of protein interaction network

**Fig. 3:**
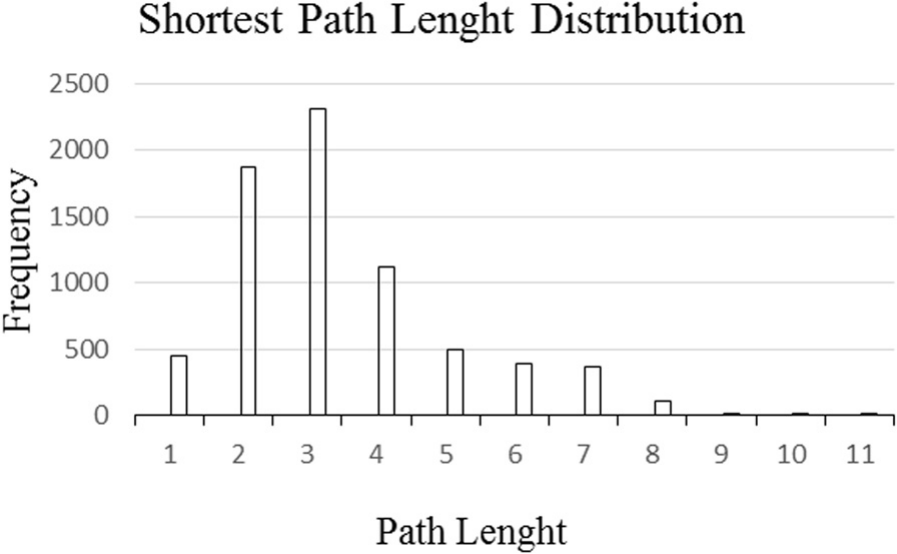
Histogram of Distribution of the Shortest Path. The shortest paths analysis of the network demonstrated that any two randomly selected nodes on the network were connected by 3.4 links. The results show that the nodes of the network were very closely linked and were similar to other deals of human protein networks

The biological networks have been represented to have a power law degree distribution, which follows scale-free topology. This important feature of biological networks is significant for a credible prediction of essentiality when the factors of connectivity and betweenness centrality are utilized. Furthermore, it is described that most of the biological networks in nature have a degree exponent amount between two and three [18, 19]. The exponent degree and R^2^ coefficient (for degree distribution) of the sperm-egg interaction network were measured using the least square method by a network analysis plugin of Cytoscape. The PPIs in the created network have been filtered for only high confidence (STRING score > 0.7 for functional and predicted interactions and MI score ≥ 0.431 for physical interactions). The created network with high confident PPIs in an exponent of −1.1 (R^2^ = 0.9) was compatibly better with a scale-free topology and also provides to assert with more reliability that a discovered hub and bottleneck node may be essential for the network [18–20]. The shortest paths analysis of the network demonstrated that any two randomly selected nodes on the network were connected by 3.4 links. The results show that the nodes of the network were very closely linked and were similar to other deals of human protein networks [19]. The distribution of the shortest paths was represented using a histogram, as shown in Fig. 3.

### Detection of the putative drug targets

**Fig. 4:**
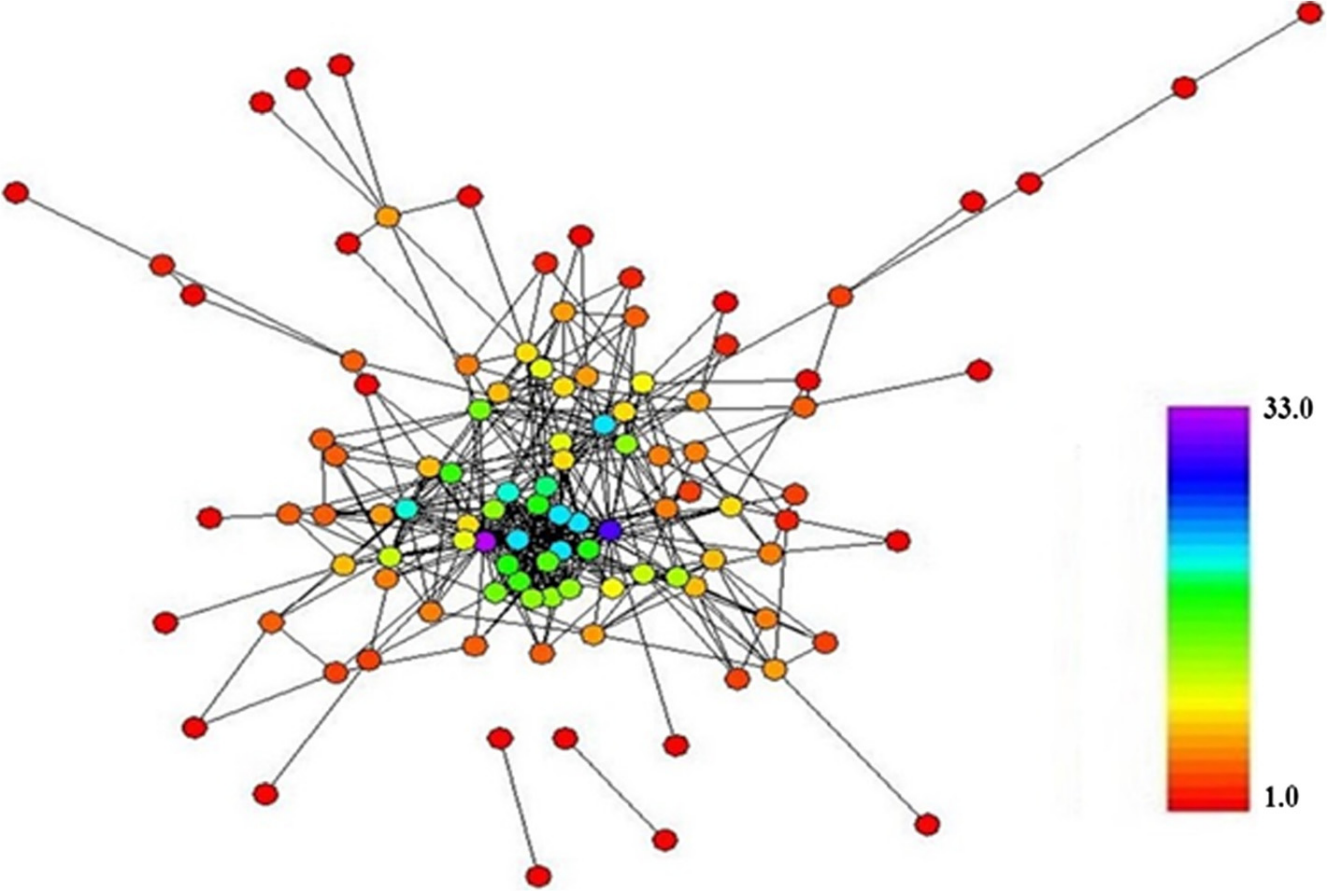
Color Filtered Sperm-Egg Interaction Network by Degree from 1–33. The degree tells us how many links a node has to other nodes. The high degree nodes as hub nodes play important roles in the survival of correlations within a biological network. If the hub nodes are attacked in a network, the network can be broken into pieces

In a scale free distribution, the presence of low number of highly connected nodes known as hubs is more significant than lesser connected nodes. The hub nodes, as represented in Fig. 4, play an important role in the survival of correlations within a biological network. If the hub nodes are attacked in a network, the network can be broken into pieces. The node with a large BC value is another important node that functions as a bottleneck in the network, even when the degree of node is low. Half of the maximum degree and BC of the network were used as the critical point of high degree and high BC nodes [18, 19, 21, 22]. In order to check topological centrality of hubs and bottlenecks in the network, CC values of the protein set were measured. The degree, BC, CC and the protein family of the important nodes are shown in Table 2.

**Table 2:** The high degree nodes with evaluated BC and CC in sperm-egg protein network

Gene name	Protein family	Degree	BC	CC
ITGB1	Integrins	33	0.2	0.5
FN1	Fibronectin	30	0.1	0.5
EGFR	Epidermal growth factor receptors	26	0.2	0.5
ITGA3	Integrins	25	0.03	0.5
ITGAV		24	0.03	0.5
ITGB3		23	0.02	0.5
COL1A1	Collagens	22	0.02	0.5
CD9	Tetraspanins	21	0.1	0.5
ITGB4	Integrins	20	0.04	0.5
ITGA5		19	0.04	0.5
ITGB8		18	0.01	0.4
ITGB5		17	0.01	0.4
ITGA2		17	0.01	0.4

Half of the maximum degree and BC of the network were used as the critical point of high degree and high BC nodes and in order to check topological centrality of hubs and bottlenecks in the network, CC values of the protein set were measured. The proteins from integrin, fibronectin, epidermal growth factor receptor, collagens and tetraspanin protein families are hub nodes that have a large BC

BC betweennes centrality, CC closeness centrality

The predominant aspect of hub and bottleneck protein nodes in a biological protein interaction network is that they are potential drug targets. If functions of hubs and bottlenecks are inhibited by small molecules, the process of the network can be stopped; on the other hand, the studying processes can be shut down, theoretically.

### Experimental evidence for the essentiality of predicted potential drug targets

**Fig. 5:**
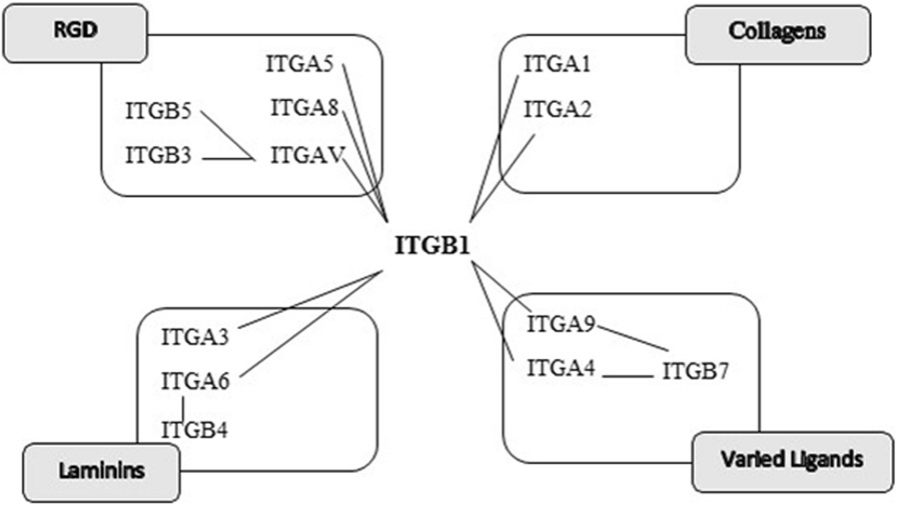
Different Types of Integrins [27]. The mouse oocytes can be expressed in diverse integrins as indicated by analysis of integrin subunit expression, i.e. ITGA9-ITGB1, laminin-binding family collagen-binding family and Arg-Gly-Asp (RGD)-binding family (such as, vitronectin, fibronectin)

The result revealed five protein families with high degree nodes within the network, as stated in Fig. 4 and Table 2. Integrins are a family of cell adhesion molecules that facilitate cell-extracellular and cell-cell matrix interactions [23, 24] that similarly have been contributed as possessing a part in association with mammalian sperm-egg interactions. Integrins are heterodimeric transmembrane proteins constituted of an “α” and a “β” subunit, including 18 α and 8 β subunits in mammals. (Mouse Genome Database and Human Genome Organization nomenclatures mention them as ITGB and ITGA subunits, respectively). Based on ligand specificity and on sequence homologies of the subunits, the 24 acknowledged heterodimer combinations are categorized into five different subfamilies [23, 25, 26]. Moreover, the ADAM (a disintegrin and metalloproteinase) is one of the most recently described groups of integrin ligands. An anti ITGB1 function-blocking antibody which decreases sperm-egg binding considerably inhibited ADAM2 adhesion to mouse oocytes. Referring to Fig. 5, the mouse oocytes can be expressed in diverse integrins as indicated by analysis of integrin subunit expression, i.e. ITGA9-ITGB1, laminin-binding family collagen-binding family and Arg-Gly-Asp (RGD)-binding family (such as, vitronectin, fibronectin). Adhesion analysis has characterized that ADAM2 interactions with ITGA9/ITGA4 family [27].

Figure 5 shows that ITGB1 can interact with other subunits, and previous studies have already demonstrated that integrin β1 was overexpressed in infertile men. *Itgb1*-deficient eggs in mice have demonstrated an interruption in sperm-egg adhesion in time-lapse video analysis, and slight deficiencies have been detected with *Itga3*-deficient eggs in modified analyses. A decrease in sperm-egg fusion and binding was shown in the oocytes with 50 % knockdown of ITGA9 on the oocyte surface as well as the reduction of the fertilized eggs percentage in comparison to the controls, but ITGA9 knockdown did not bring a comprehensive loss of fertilization ability. This occurred possibly due to a partial decrease of ITGA9 on the oocyte surface and because of likely function of other molecules on the oocyte surface in gamete membrane adhesion [28, 29]. However, oocyte integrins, mainly the ITGA9 α subunit, possess the prospect to contribute to sperm-oocyte interactions with wild-type oocytes, although data from knockouts establish that oocytes lacking in several integrins are still able to be fertilized. The presented result showed that ITGA9 interacts with ITGB1 as a hub protein in the created human sperm-egg interaction. Due to previous studies, the incubation of bull spermatozoa with integrin beta 5 antibodies considerably reduced the capability of fertilizing the oocyte. The results have recommended that during fertilization, the bovine sperm integrin beta 5 protein plays an important role and could serve as a functional or a positional marker of bull fertility [30]. The CD9 in the network is a hub node to facilitate the sperm-egg interaction. CD9 and other tetraspanins are possibly taking part in the adjustment of membrane directed by connecting to and/or enabling the acting of other membrane proteins. CD9-associated proteins in other cell categories are comprised of IgSF members, numerous integrins and other adhesion molecules, ectoenzymes and several intracellular signalling molecules. However, there is only restricted information regarding CD9-associated proteins in oocytes. IgSF8 co immune precipitates withCD9 in oocyte lysates and is lacking from the *Cd9*-null eggs’ surfaces [3, 31].

Fibronectin (FN) binds to the cell surface via integrins, which are transmembrane protein receptors. The integrins identify the Arg-Gly-Asp (RGD) amino acid sequence in FN. Following capacitation, the human sperm express FN on their surface, and FN is also secreted during cumulus penetration. Association of FN and its α5β1 integrin receptor with IVF has previously been revealed in a number of species [32, 33].

Furthermore, FN is a constituent of the seminal fluid and a ubiquitous multifunctional glycoprotein that plays an important role in the formation of seminal gel after ejaculation. It can bind to cellular components that are visible once spermatozoa is impaired and consequently assists in selecting damaged spermatozoa [34, 35]. An increase in fibronectin expression of infertile patients compared to fertile cases has been reported [36].

Epidermal growth factor receptors (EGFRs) are receptor tyrosine kinases and are activated by a big family of peptidic ligands that persuade the constitution of active auto (trans)-phosphorylated receptor homo-/heterodimers. Upon recruitment of signalling proteins and adaptors, the active dimers start multiple signalling procedures [37]. It has been shown previously that bovine sperm comprises EGFR, localized to the sperm head and the mid-piece. Also, it has been revealed that during sperm capacitation, EGFR is involved in the AR [38–40]. In addition, EGFR phosphorylation/activation is increased during capacitation. While further stimulation of the EGFR in capacitated sperm reveals augmented intracellular calcium amounts, leading to AR [38]. A scan of the microarray device showed that zona pellucida glycoprotein 3 (ZP3) interacted with EGFR, and EGFR is a new suggested sperm receptor for ZP3. EGFR-null sperm show very low binding to the egg as well as reduced IVF rate, emphasizing the role of EGFR and its necessity for efficient sperm-egg interaction for normal fertilization [41].

Collagens are members of the extracellular matrix proteins family that plays a significant role in developing processes and are essential structural components of organs and tissues, comprising skin, lung, blood vessels and bone. A key collagenous constituent, Type I collagen, functions in neural crest cell migration and may be linked to the regulation of cellular variation. Col1a1 have been recognized from published microarray experiments on normal human tissue [33, 42, 43], and there is a high level of expression in reproductive tissues. Col1a1 is greatly stated in the uterus and plays a significant role during spermatogenesis where it facilitates the migration and detachment of germ cells. The function of procollagen I in germ cells is associated with their ability to attach and detach from the basement membrane, and it seems that during spermatogenesis, procollagen I might be included in regulating the connection of these germ cells to the basement membrane inside the seminiferous tubules. The damage of procollagen I expression may announce a beginning of successive differentiation into later germ cell kinds [33].

Integrins are membrane receptors that link cells to the extracellular matrix, comprising collagen, and function in regulating proliferation, cellular migration, survival and differentiation. β_1_ integrin can make couple with various α-subunits to make two main receptors for type I collagen; in particular, α_1_β_1_ and α_2_β_1_ integrins are efficient cellular receptor for type I collagen [44].

### Detection of drugs for the putative drug targets

The known drugs for the identified drug targets could be found using IPA as represented in Table 3. In Table 3, the targets were arranged based on their degree in the sperm-egg interaction network from high to low. IPA’s data analysis and search capabilities show the significance of the yield data, specific target, or candidate biomarkers in the context of larger biological or chemical systems.

**Table 3:** Known drug targets and drugs for the candidate protein in sperm-egg protein interaction Network

Protein name	Drugs	Degree
FN1	Ocriplasmin	30
EGFR	Cetuximab, AEE 788, panitumumab, BMS-599626, varlitinib, XL647, afatinib, sapitinib, cetuximab/irinotecan, erlotinib/gemcitabine, lapatinib/letrozole, canertinib, gefitinib, neratinib, PD 153035, lapatinib, vandetanib, erlotinib	25
ITGAV	Abciximab, CNTO 95, cilengitide	24
ITGB3	Abciximab, TP 9201, cilengitide, tirofiban	23
COL1A1	Collagenase clostridium histolyticum	22
ITGB5	Cilengitide	17

The identified drug targets were arranged based on their degree in the sperm-egg interaction network from high to low. The known drugs for the targets were found using IPA

As discussed in the topological analysis of PINs, the high degree and high BC nodes in PPI networks from the complex diseases are the putative drug targets for future studies in diagnoses or medical treatments. Topological analysis of the sperm-egg protein interaction network have been showed that FN1, EGFR, ITGAV, ITGB3, COL1A1, ITGB5 have high degree and BC, and it is assumed these proteins are putative drug targets for future medical studies in treatment of hidden male infertility or unsuccessful ART.

Increased expression of fibronectin has been reported in infertile patients when compared with fertile cases [36]. Fn and the integrin receptors for Fn (α5β1) in humans exist on the spermatozoa surface after capacitation, signifying that accumulated exogenous Fn perhaps compete with those Fn molecules stated on the surface of sperm in binding receptors on the ZP. Plasma Fn as exogenous Fn lacks the fragment which includes a second RGD sequence and intrinsically cannot function as a sperm-oocyte linking molecule. A second RGD sequence is essential in order to apply this kind of velcro function, and this second RGD sequence has a role in several biological substitutes of Fn that are positioned on the zona pellucida [45].

By combining the previously represented outcomes, it can be presumed that the over expression of plasma FN1 may inhibit the interaction of the endogenous FN ligands with corresponding receptors on both sperm cells and the *oocyte* plasma *membrane*. Until now, only “Ocriplasmin (Jetrea©)” has been used to degrade fibronectin. Ocriplasmin is a recombinant protease with activity against fibronectin that is used for treatment of symptomatic vitreomacular adhesion. However, the relationship between Ocriplasmin and fertility has not been considered, and further studies need to design drugs that can induce plasma FN1 degradation in over expressed conditions.

Additionally, a scan of the microarray device displayed that ZP3 interacted with EGFR which is a new suggested sperm receptor for ZP3. On the other hand, EGFR-null sperm revealed very low binding to the oocyte as well as decreased IVF rate, emphasizing the role of EGFR and its essentiality for efficient sperm-egg interaction for normal fertilization [41]. Amplification or overexpression of EGFR has been revealed to play a significant role in the progression and development of certain aggressive types of breast cancer [46]. Although no medical relationship between overexpress of EGFR and infertility has been reported, Meggiorini and his co-workers have already established that female patients with primary infertility may symbolize a group at greatest risk for breast cancer [47]. Combining these results, it is assumed that overexpression of EGFR might be a potential cause of infertility.

EGFR has been identified as an oncogene that led to the progression of anticancer therapeutics directed against EGFR (called “EGFR inhibitors”). Many drugs against EGFR are monoclonal antibody inhibitors which block the extracellular ligand binding domain. Therefore, the tyrosine kinase cannot be activated due to no attachment of signal molecules. Cetuximab (Erbitux©) and panitumumab (Vectibix©) are examples of monoclonal antibody inhibitors [48]. Other types of drugs against EGFR are small molecules to inhibit the EGFR tyrosine kinase which is on the cytoplasmic side of the receptor. Accordingly, EGFR is unable to activate itself, and by stopping the signaling cascade in cells that is based on this pathway, growth, migration and proliferation of tumor will be diminished. Gefitinib (Iressa©) and erlotinib hydrochloride (Tarceva©) (mixed EGFR and ERBB2 inhibitor) are examples of small molecule kinase inhibitors [49]. These drugs with EGFR inhibitor functions are suggested to study their effects on infertile cases that present overexpression of EGFR.

The integrins are membrane receptors that function in regulating proliferation, cellular migration, survival and differentiation and also link cells to the extracellular matrix comprising collagen. In order to constitute two main receptors for type I collagen, β_1_ integrin can couple with diverse α-subunits, namely α_1_β_1_ and α_2_β_1_ integrin [44].

According to the result of this study, β_1_ integrin (ITGB1) has the highest degree in the sperm-egg interaction network and previously has been detected in both sperm and egg [50]. The combining results show that the type I collagen (COL1A1) induces the binding between sperm and oocyte through interaction with β_1_ integrin receptors in both cells. COL1A1 is the target for the Collagenase clostridium histolyticum (Xiaflex©) drug. Collagenase clostridium histolyticum is an enzyme generated by the bacterium *Clostridium histolyticum* that pull apart collagen. It is used in the form of a powder-and-solvent injection kit for the treatment for Dupuytren’s contracture, an illness in which the fingers twist towards the palm and cannot be completely flattened [51, 52]. Recently this medicine has been approved by FDA for males [53]. Due to the effect of this drug in treatment these diseases, any relationship between this drug and sperm-egg interaction cannot be found.

Overexpression of some type of integrins, such as ITGB3, ITGB5 and ITGAV, has been associated with the tumor metastasis in breast cancer. Targeting these cell surface proteins could provide a possible assay to consider the metastatic potential of tumors [54]. Additionally, it has been reported that with primary infertility, female patients might symbolize a group at great danger of breast cancer [47]. The results of this study also show the molecular aspects of the link between sperm-egg disorder and breast cancer. Combining these result, the up regulation of ITGB3, ITGB5 and ITGAV could be assumed may disturb sperm-egg binding and fusion and fail successful fertility. Clingitide seems to inhibit tumor regression by inhibiting these integrins [55, 56]. Therefore, if the relationship between the up-regulated integrins and sperm-egg binding perturbation are supported by further experimental work, Clingitide could be an interesting drug for future treatment of sperm-egg binding disorder.

The roles of the mentioned drug targets on sperm-egg interaction and the hypothetical effects of the drugs on treatment of sperm-egg interaction defects are summarized in Table 4.

**Table 4:** Hypothetical effects of the targets’ drugs on treatment of sperm-egg interaction defects

Protein name	Role in sperm-egg interaction	Expression level/infertile vs fertile	Hypothetical role of drugs	Drug name/effect	Suggest for further study on sperm-egg interaction
FN1	Interact with α5β1 in sperm and oocyte	↑ [37]	↑ FN1 may defect sperm-egg interaction	Ocriplasmin/↓	√
EGFR	Interact with ZP3	↑ in breast cancer and there is a relationship between breast cancer and infertility [48]	↑ EGFR may defect sperm-egg interaction	Cetuximab, Panitumumab, Gefitinib, erlotinib hydrochloride/ ↓	√
COL1A1	Interact with β1 in sperm and oocyte	×	Induces the binding sperm-egg interaction	Collagenase clostridium histolyticum/ pull apart	×
ITGAV, ITGB3, ITGB5	Facilitates interaction between sperm and egg	↑ in breast cancer [55] and there is a relationship between breast cancer and infertility [48]	↑ ITGAV, ITGB3, ITGB5 may defect sperm-egg interaction	Cilengitide/↓	√

The roles of the mentioned drug targets on sperm-egg interaction and the hypothetical effects of the drugs on treatment of sperm-egg interaction defects have been represented in Table 4

↑ increase

↓ decrease

## Conclusion

This study constitutes the first attempt to determine a potential list of drug targets for human sperm-egg interaction defects using a protein interaction network approach. The detected targets are from integrins, fibronectins, epidermal growth factor receptors, collagens and tetraspanins protein families. The known drugs for the identified targets have been identified using IPA, and the possible roles of the drugs on sperm-egg interaction have been discussed. These results showed that the drugs ocriplasmin (Jetrea©), gefitinib (Iressa©), erlotinib hydrochloride (Tarceva©), clingitide, cetuximab (Erbitux©) and panitumumab (Vectibix©) are possible candidates for efficacy testing on human sperm-egg interaction disorder for further experimental validation. This strategy, if validated, may develop established drug discovery methodologies and also can be helpful for assisted reproductive technology to avoid IVF failure with a remarkable reduction in investment of time and resources.

## Additional file

Additional file 1: Table S1. The Physical PPIs in Sperm-Egg Interaction Network. **Table S2.** The Functional and Predicted PPIs from GeneMANIA Database in Sperm-Egg Interaction Network.

## Abbreviations

IVF: *In-vitro* fertilization
IPA: Ingenuity pathway analysis
PPI: Protein-protein interaction
PIN: Protein interaction network
BC: Betweenness centrality
CC: Closeness centrality
ADAM: A disintegrin and metalloproteinase
FN: Fibronectin
EGFRs: Epidermal growth factor receptors
ZP3: Zona pellucida glycoprotein 3
RGD: Arg-Gly-Asp
