# Supplementary material for: Identification of putative drug targets for human sperm-egg interaction defect using protein network approach
Source: BMC Syst Biol. 2015 Jul 18;9:37. doi: 10.1186/s12918-015-0186-7 (PMC4506605; doi:10.1186/s12918-015-0186-7)
Supplement: Additional file 1: Table S1. — The Physical PPIs in Sperm-Egg Interaction Network. Table S2. The Functional and Predicted PPIs from GeneMANIA Database in Sperm-Egg Interaction Network. [file 12918_2015_186_MOESM1_ESM.pdf]

**Additional file**

**Table S1:** The Physical PPIs in Sperm-Egg Interaction Network

| Feature Category | Database | No. of Interactions |
|---|---|---|
| **Physical Interaction** | DIP | 7 |
| | BIND | 33 |
| | HPRD | 172 |
| | IntAct | 13 |
| | MINT | 9 |
| | MIPS | 6 |
| | APID | 230 |
| | BioGRID | 138 |
| | | Total: 239 |

**Table S2:** The Functional and Predicted PPIs from GeneMANIA Database in Sperm-Egg Interaction Network

| Feature Category | Reference | Number of PPI |
|---|---|---|
| **Co-expression** | Gene Expression Omnibus (GEO) (Barrett et al., 2013) | 793 |
| **Genetic interaction** | BioGRID (Stark et al., 2006) | 333 |
| **Co-localization** | Gene Expression Omnibus (GEO) (Barrett et al., 2013), Gene-Ontology (GO) (Carbon et al., 2009) | 190 |
| **Pathway** | Reactome (Vastrik et al., 2007), BioCyc (Karp et al., 2005), PathwayCommons (Cerami et al., 2011) | 224 |
| **Shared protein domains** | InterPro (Apweiler et al., 2000), SMART (Letunic et al., 2012), Pfam (Bateman et al., 2004) | 178 |
| | | Total: 1399 |
